# To treat or not to treat: a comparative effectiveness analysis of oral anticoagulant outcomes among U.S. nursing home residents with atrial fibrillation

**DOI:** 10.1186/s12877-024-05186-9

**Published:** 2024-07-19

**Authors:** Qiaoxi Chen, Jonggyu Baek, Robert Goldberg, Jennifer Tjia, Kate Lapane, Matthew Alcusky

**Affiliations:** 1 Population Health Sciences Program, Morningside Graduate School of Biomedical Sciences, University of Massachusetts Chan Medical School, Worcester, MA 01655 USA; 2 Division of Epidemiology, Department of Population and Quantitative Health Services, University of Massachusetts Chan Medical School, 55 N Lake Ave, Worcester, MA 01655 USA

**Keywords:** Comparative effectiveness, Safety, Oral anticoagulants, Nursing home

## Abstract

**Background:**

Nursing home residents with atrial fibrillation are at high risk for ischemic stroke, but most are not treated with anticoagulants. This study compared the effectiveness and safety between oral anticoagulant (OAC) users and non-users.

**Methods:**

We conducted a new-user retrospective cohort study by using Minimum Data Set 3.0 assessments linked with Medicare claims. The participants were Medicare fee-for-service beneficiaries with atrial fibrillation residing in US nursing homes between 2011 and 2016, aged ≥ 65 years. The primary outcomes were occurrence of an ischemic stroke or systemic embolism (effectiveness), occurrence of intracranial or extracranial bleeding (safety) and net clinical outcome (effectiveness or safety outcomes). Secondary outcomes included total mortality and a net clinical and mortality outcome. Cox proportional hazards and Fine and Grey models estimated multivariable adjusted hazard ratios (aHRs) and sub-distribution hazard ratios (sHRs).

**Results:**

Outcome rates were low (effectiveness: OAC: 0.86; non-users: 1.73; safety: OAC: 2.26; non-users: 1.75 (per 100 person-years)). OAC use was associated with a lower rate of the effectiveness outcome (sHR: 0.69; 95% Confidence Interval (CI): 0.61–0.77), higher rates of the safety (sHR: 1.70; 95% CI: 1.58–1.84) and net clinical outcomes (sHR: 1.20; 95% CI: 1.13–1.28) lower rate of all-cause mortality outcome (sHR: 0.60; 95% CI: 0.59–0.61), and lower rate of the net clinical and mortality outcome (sHR: 0.60; 95% CI: 0.59–0.61). Warfarin users, but not DOAC users, had a higher rate of the net clinical outcome versus OAC non-users.

**Conclusions:**

Our results support the benefits of treatment with OACs to prevent ischemic strokes and increase longevity, while highlighting the need to weigh apparent benefits against elevated risk for bleeding. Results were consistent with net favorability of DOACs versus warfarin.

**Supplementary Information:**

The online version contains supplementary material available at 10.1186/s12877-024-05186-9.

## Background

Anticoagulants are effective in preventing atrial fibrillation related cardioembolic complications and are recommended for patients at high risk for stroke [1–7]. However, oral anticoagulant (OAC) use is associated with an increased risk of bleeding [8–10]. Clinical trials have provided evidence of the comparative effectiveness and safety between warfarin versus aspirin, and between direct oral anticoagulants (DOACs) and warfarin, in patients with atrial fibrillation [11–18]. OACs have been found to be superior for stroke prevention when compared with aspirin, while DOACs have been either non-inferior or superior in terms of stroke prevention and risk of bleeding, when compared with warfarin [12–15, 19]. Observational studies have confirmed the effectiveness and safety of DOACs as compared with warfarin, particularly in community-dwelling populations [20–30].

The risk-benefit profile of OAC use has not been thoroughly examined among nursing home residents. Nearly all nursing home residents with atrial fibrillation are indicated for anticoagulation due to their advanced age and strong clinical indications for being at high risk for stroke, though OAC use in this population has remained below levels seen in the outpatient setting [31]. The lower use of OACs in the nursing home setting reflects concerns about whether to treat these individuals due to a perceived smaller net clinical outcome of oral anticoagulation among older adults with multiple comorbidities and competing mortality risk [32]. Physician and facility related factors also contribute to OAC prescribing decisions in nursing home residents [33, 34].

Weighing the benefits of ischemic stroke prevention against increased bleeding risk for older nursing home residents is challenging, since evidence specific to this care setting is sparse. Treatment goals often shift away from consensus clinical guidance adherence, which are more appropriate for younger community-dwelling populations, and towards individualized care goals. Therefore, to generate evidence specific to the nursing home setting to better inform clinical decision-making, this study examined the effectiveness and safety of OAC use among older nursing home residents with atrial fibrillation using a national data resource.

## Methods

This study was approved by the University of Massachusetts Chan Medical School Institutional Review Board, which issued a consent waiver for this study.

### Data sources

We used the Minimum Data Set 3.0 (MDS 3.0) linked with Medicare administrative files. The MDS 3.0 contains resident-level information from federally mandated assessments (MDS assessments) routinely conducted every 3 months in all Medicare/Medicaid-certified nursing homes of functional status, cognitive impairment, medical diagnoses, treatments received, and behavioral symptoms. The Master Beneficiary Summary File contains demographic and eligibility information for Medicare beneficiaries. The Medicare fee-for-service Part A database contains service dates, clinical diagnoses, and procedure codes from hospitals and skilled nursing facilities (SNF). The Medicare Part D drug characteristics and drug event files provide individual-level prescription information.

### Study design

We conducted a retrospective cohort study of U.S. nursing home residents ≥ 65 years diagnosed with atrial fibrillation to compare effectiveness and safety outcomes between OAC new users versus non-users. Primary analyses compared the time to three composite endpoints (a primary effectiveness outcome, a primary safety outcome, and a net clinical outcome with death as a competing risk) between these groups. We compared time to death and time to a net clinical and mortality outcome as secondary outcomes between OAC users and non-users. We performed stratified analyses among warfarin users and direct-acting oral anticoagulants (DOACs) users versus non-users, respectively. All analyses were based on an as-treated study design.

### Study population

Inclusion criteria of our study population were: (1) residence in a U.S. Medicare or Medicaid certified nursing home (not a SNF stay) at the time of their index date (see below); (2) continuous enrollment in Medicare fee-for-service Part A and Part D for the 6-month baseline period; and a (3) diagnosis of atrial fibrillation or flutter (without any valvular diseases) according to MDS assessment or inpatient claims (Supplemental Table 6) during the 6-month baseline period; (4) aged 65 or more. We excluded residents who died on or before the index date, and those who were comatose or who were in hospice as recorded on their most recent MDS assessment before the index date.

### OAC use and non-use

For the new OAC user group, we included U.S. nursing home residents ≥ 65 years who had a first OAC prescription between July 1, 2011 and December 31, 2016 to ensure a minimum 6-month baseline period (Supplemental Fig. 1). The first OAC prescription date was defined as the resident’s index date among OAC users.

For the non-user comparison group, we included older adults who did not have an OAC prescription according to Part D claims during the 6-month period before their index MDS assessment date. Among all eligible MDS assessments of non-users occurring on or after July 1 2011 (to ensure a minimum 6-month baseline), we randomly chose one assessment date as their index date. (Supplemental Fig. 2). To reduce the potential for selection bias associated with selecting a comparison group of residents who never used OACs, we allowed OAC users to be included in the non-user group if they had any MDS assessments where eligibility for the non-user group was met.

Among the 476,835 nursing home residents aged ≥ 65 years with atrial fibrillation, 37,107 were OAC users and 443,484 were non-users; 3,756 residents contributed person-time to both the OAC user and non-user groups.

### Study outcomes

The principal study outcomes were the time from the index date until the occurrence of any component of the primary composite effectiveness outcome, the primary safety composite outcome, and the net clinical outcome. The primary composite effectiveness outcome was development of an acute ischemic stroke or systemic embolism defined by ICD-9 or ICD-10 codes in Medicare Part A hospital claims (Supplemental Table 6). The composite primary safety outcome was the occurrence of an intracranial or extracranial hemorrhage. The net clinical outcome was a composite of the primary effectiveness and safety outcomes. The secondary study outcomes included the time to the earliest occurrence of a net clinical and mortality outcome and time to death. The net clinical and mortality outcome included the effectiveness composite outcome, safety composite outcome, and death.

We followed residents for up to two years or until a study outcome, death (a competing risk), or a censoring event occurred. Censoring events included a change in OAC use status (i.e., OAC discontinuation among users or OAC initiation among non-users), end of Medicare fee-for-service Parts A and D coverage, and end of the study period. OAC users were not censored for switching between DOACs and warfarin. We set the OAC discontinuation date at the end of a 14-day gap in treatment or 14 days after the end date of the last prescription.

### Covariates

Demographic covariates included age, sex, and race/ethnicity. Race/ethnicity was categorized using information from the MDS. Body mass index (BMI) was derived from the MDS [35]. We used information from the most recent MDS assessment, and Part A claims and/or Part D claims occurred during the six months before the index date to construct the following variables. CHADs-VASc ischemic stroke risk scores were categorized as 0–1, 2–4, 5–6, and ≥ 7 [36]. The Anticoagulation and Risk factors in Atrial Fibrillation (ATRIA) bleeding risk score was grouped into 0–3 points (low risk), 4 points (intermediate risk), and 5–10 points (high risk) [37]. We identified recent hospitalizations during the baseline period due to ischemic stroke, venous thromboembolism, systemic embolism, acute myocardial infarction, intracranial hemorrhage, and extracranial hemorrhage and whether residents underwent inpatient surgery. Other potential confounders included a history of recent falls, cancer diagnosis, rejection of medical care, and a diagnosis of Alzheimer’s disease and related dementia, hypertension, heart failure were obtained from Medical Part A crosslinked with MDS 3.0. Cognitive status was based on the Cognitive Function Scale with four levels: cognitively intact, mild, moderate, or severe cognitive impairment [38]. The activities of daily living (ADL) score was categorized as either low functional dependency (0–7), mild functional dependency (8–14), moderate functional dependency (15–21), and high functional dependency (22–28) [39]. We included as covariates baseline use of non-steroidal anti-inflammatory drugs (NSAIDs), antiplatelets, statins, and selective serotonin reuptake inhibitors (SSRI), which may potentially interact with OAC use or affect our study outcomes [40–42]. We counted the number of unique generic names from all baseline prescriptions and categorized three levels of polypharmacy (0–5, 6–10, and > 10).

### Data analysis

We first described the baseline characteristics of new OAC users and non-users and reported standardized mean differences (SMD). Then we performed crude and multivariable adjusted Fine and Gray regression models [43] to estimate subdistribution hazard ratios (sHRs) for the primary outcomes accounting for death as a competing risk. We used Cox Proportional models to estimate crude and multivariable adjusted hazard ratios (aHR) for time to the secondary study outcomes, which did not have competing a risk of death.

To examine the robustness of the findings from our primary analyses to potential unmeasured confounding, we performed an instrumental variable (IV) analysis. Nursing home level prescribing preference for OAC use served as the IV [44]. (Supplemental Fig. 3) Detailed steps in estimation, testing of assumptions, and outcome modeling for the IV analysis are in the Technical Appendix.

We performed stratified analyses: (1) by OAC class initiated (DOACs or warfarin), (2) the years 2011–2013 and 2014–2016 to examine potential implications of DOAC channeling in the early years after medication market entry; (3) by the highest levels of stroke and bleeding risk, highest level of stroke risk only, highest level of bleeding risk only, and lower levels of stroke and bleeding risk; and (4) by antiplatelet use.

### Role of the funding source

The study was funded by the National Institute on Aging (R21AG060529-01). The funder had no involvement in the study.

## Results

### Study population characteristics

The median age among the 37,107 OAC users was 83 and four years younger than the 443,484 non-users (Table 1). Approximately two-thirds of OAC users and non-users were women and the approximately 20% of OAC users and non-users had CHADS_2_-VASc score with ≥ 7. Age and several clinical covariates had SMDs > 0.10 indicating the distributions were meaningfully different for users and non-users.

**Table 1:** Characteristics of nursing home residents with atrial fibrillation (*N* = 480,591)*

	OAC new users (*N* = 37,107)	OAC non-users (*N* = 443,484)	Standardized mean difference^b^
**Age**			
Median, years (25th, 75th percentile)	83 (76, 89)	87 (80, 92)	0.38^b^
**Women, %**	66.8	67.3	0.009
**Race/Ethnicity, %**			
Non-Hispanic White	82.6	85.1	0.07
Black/African American	12.2	9.9	0.07
Hispanic	2.2	1.9	0.02
American Indian/Alaska Native	0.4	0.4	0.006
Asian/Pacific Islander	1.5	1.7	0.02
Races not specified above	0.8	0.8	0.01
Body Mass Index (BMI)^*^, **kg/m**^**2**^			
Median (25th, 75th percentile)	27.2 (23.2, 32.4)	24.6 (21.1, 28.9)	0.009
**Comorbidities, %**			
Heart failure	47.4	42.5	0.12^b^
Hypertension	80.4	85.8	0.15^b^
Diabetes mellitus	44.4	36.9	0.10
Stroke	26.4	20.5	0.09
Coronary artery disease	43.2	42.8	0.43^b^
Venous thromboembolism	15.8	2.9	0.11^b^
Peripheral vascular disease	16.3	16.0	0.13^b^
Anemia	30.8	40.7	0.21^b^
End-stage renal disease	9.5	17.9	0.25^b^
Dialysis	3.3	3.3	0.003
Liver disease	2.6	2.3	0.02
Cancer	11.8	13.8	0.35^b^
History of Fall	35.7	39.4	0.08
Alzheimer’s disease and related dementias	51.3	60.7	0.19^b^
**Rejects care, %**	16.5	7.1	0.29^b^
**Hospital admission in prior year, %**			
0	29.1	58.3	0.62 ^b^
1	37.5	22.5	0.33 ^b^
≥2	33.4	19.2	0.33 ^b^
**Reasons for hospitalization in the six months prior to index date, %**			
Ischemic stroke hospitalization	6.8	2.1	0.23^b^
Transient ischemic attack	1.0	0.4	0.07
Stroke	9.0	3.2	0.11^b^
Intracranial bleed	0.3	0.4	0.03
Extracranial bleed	2.1	2.3	0.02
Acute myocardial infarction	3.3	2.0	0.08
**Hospitalization with inpatient surgery in past 6 months, %**	44.1	24.4	0.42^b^
**SNF admission in prior year, %**			
0	67.7	79.9	0.28^b^
1	20.4	12.8	0.21^b^
≥2	11.9	7.3	0.16^b^
**CHA**_**2**_ **DS**_**2**_ **-VASc Risk Score, %**			
≥ 7	21.5	17.1	0.05
5–6	46.4	47.0	0.06
2–4	31.8	35.6	0.10
0–1	0.3	0.4	0.02
**ATRIA score, %**			
High risk (5–10)	44.4	49.3	0.10
Intermediate risk (4)	7.2	4.4	0.12^b^
Low risk (0–3)	48.3	46.3	0.04
**Moderate/severe cognitive impairment, %**	33.9	48.6	
Moderate or severe	33.9	48.6	0.21^b^
Mild/none	66.1	51.4	0.03
**Activities of daily living**^**a**^, **%**			
High functional dependency	13.9	21.0	0.19^b^
Moderate functional dependency	44.3	45.1	0.02
Mild functional dependency	27.0	22.5	0.11^b^
Low functional dependency	14.9	11.4	0.10
**Nonsteroidal anti-inflammatory drug (NSAID)**	16.1	13.1	0.08
**Antiplatelets**	18.7	17.9	0.02
**Statins**	47.8	39.6	0.17^b^
**Non-selective beta blockers**	4.9	< 0.1	0.32^b^
**Beta blockers indicated for heart failure**	45.6	38.8	0.14^b^
**Other selective beta blockers**	21.1	18.2	0.07
**Antiarrhythmics (Class I and Class III)**	22.8	19.1	0.09
**Cardiac glycosides**	14.4	13.3	0.03
**ACE inhibitor or ARB**	48.0	41.0	0.14^b^
**Diuretics**	57.2	50.1	0.14^b^
**Polypharmacy, %**			
Using ≥ 11 medications	68.1	57.3	0.22^b^
Using 6–10 medications	23.9	30.5	0.15^b^
Using ≤ 5 medications	8.1	12.2	0.14^b^

^*^ All “N” numbers were documented as number of records Users: ADL has 25 missing records; BMI has 531 missing records

^a^ Non-users: ADL has 535 missing records; BMI has 12,044 missing records

^b^ Standard Mean Difference (SMD): covariates that had absolute SMD > 0.10

### Primary study outcomes

The median follow-up time to the earliest occurrence of the composite effectiveness outcome of ischemic stroke or systemic embolism was 348 days among OAC users and 193 days among non-users. The median duration of follow-up until a first intracranial or extracranial hemorrhage was 169 days and 155 days among OAC users and non-users, respectively. The incidence rate of the primary effectiveness outcome was markedly lower in OAC users as compared with non-users, whereas the incidence rate of the primary safety outcome was higher in OAC users versus non-users. The incidence rate of the net clinical outcome was slightly lower in OAC users and non-users, respectively (Table 2).

**Table 2:** Estimated incidence rates for primary and secondary study outcomes among OAC users and non-users, and crude and adjusted subdistribution hazard ratios (SHR) for primary outcomes in Fine and Gray models, hazard ratio (HRs) for secondary outcomes in Cox proportional hazard models comparing OAC users and non-users (*N* = 480,591)

	OAC users		OAC non-users			
	% with event	Incidence Rate (per 100 person-years)	% with event	Incidence Rate (per 100 person-years)	Crude SHR*	Adjusted SHR*
**Primary Effectiveness Outcome**	0.9	0.86 (0.77–0.96)	1.3	1.73 (1.68–1.77)	0.66 (0.59–0.74)	0.69 (0.61–0.77)
**Primary Safety Outcome**	2.3	2.26 (2.11–2.42)	1.3	1.75 (1.71–1.80)	1.73 (1.61–1.86)	1.70 (1.58–1.84)
**Net Clinical Outcome**	3.2	3.14 (2.96–3.32)	2.7	3.50 (3.44–3.56)	1.20 (1.13–1.27)	1.20 (1.13–1.28)
					Crude HR*	Adjusted HR*
**Death**	44.2	42.64 (41.99–43.29)	61.1	78.96 (78.66–79.26)	0.57 (0.56–0.58)	0.60 (0.59–0.61)
**Net Clinical and Mortality Outcome**	44.8	43.76 (43.10- 44.43)	61.5	80.41 (80.10- 80.71)	0.58 (0.57–0.59)	0.60 (0.59–0.61)

*Subdistribution hazard ratios (SHRs) estimated from Fine and Gray models. Hazard ratios (HR) estimated from Cox proportional hazards models

**Fig. 1:**
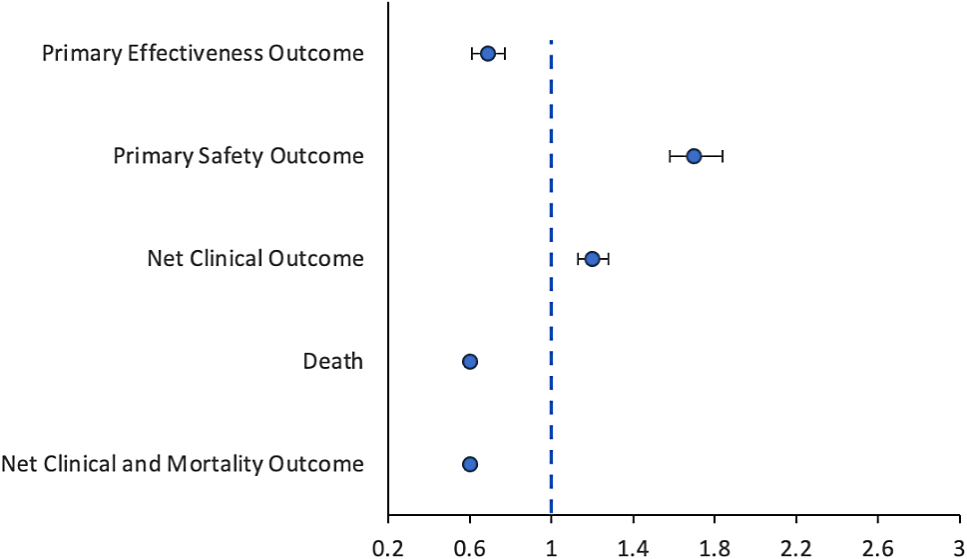
Multivariable adjusted subdistribution hazard ratios^*^ and hazard ratios^*^ for primary andsecondary outcomes comparing OAC users versus OAC non-users in Fine and Gray Models and Cox proportionalhazard models (*N* = 480,591)

In Fine and Grey models, OAC use was associated with a lower rate of the primary effectiveness outcome (aHR: 0.69; 95% CI: 0.61–0.77), higher rate of the primary safety outcome (aHR: 1.70; 95% CI: 1.58–1.84), and higher rate of the net clinical outcome (aHR: 1.20; 95% CI: 1.13–1.28) (Table 2; Fig. 1).

### Secondary study outcomes

The all-cause mortality rate was around 50% lower among OAC users and non-users, respectively, while the rate of the net clinical and mortality outcome was markedly lower among OAC users than non-users (Table 2).

In multivariable adjusted Cox proportional hazard models, OAC use was associated with lower mortality (aHR: 0.60; 95% CI: 0.59–0.61) and net clinical and mortality outcome rates (aHR: 0.60; 95% CI 0.59–0.61) (Table 2; Fig. 1).

### Results of instrumental variable analyses

**Fig. 2:**
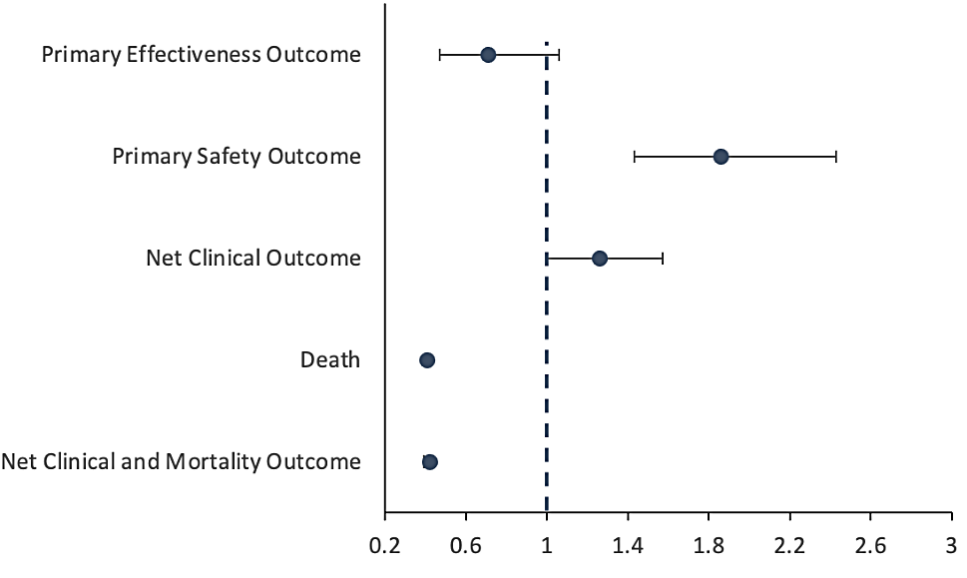
Multivariable adjusted subdistribution hazard ratios^*^ and hazard ratios^*^ for primary and secondary outcomes comparing OAC users versus OAC non-users in two-stage instrumental variable (IV)^a^ Models^b^ (*N* = 258,809)

Results from our instrumental variable analyses were generally consistent with and supported the robustness of the results of our main analyses. (Supplemental Table 1, Fig. 2)

### Stratified analyses

The crude and multivariable adjusted HRs for the primary and secondary study outcomes comparing warfarin users versus OAC non-users, and those comparing DOAC users versus OAC non-users, were similar to the primary analyses (Table 3). The net clinical outcome rate was higher among warfarin users, but not DOAC users, compared with non-users.

**Table 3:** Estimated prevalence, incidence rate, crude and adjusted subdistribution hazard ratios (SHR) for primary outcomes and hazard ratios for secondary outcomes betw﻿een warfarin users and DOAC users versus OAC non-users

	% of events among users/non-users	Incidence Rate among warfarin users (per 100 person-years)	Fine and Gray model	
			Crude SHR	Adjusted SHR
**Warfarin (** ***n*** ** = 467,283)**				
Primary Effectiveness Outcome	1.0/1.3	0.86 (0.75–0.98)	0.68 (0.59–0.77)	0.71 (0.62–0.81)
Primary Safety Outcome	2.7/1.3	2.41 (2.23–2.61)	1.90 (1.75–2.06)	1.85 (1.69–2.01)
Net Clinical Outcome	3.6/2.7	3.29 (3.07–3.51)	1.29 (1.20–1.38)	1.29 (1.21–1.39)
			**Cox proportional hazard model**	
			**Crude HR**	**Adjusted HR**
Death	48.3/61.1	43.06 (42.28–43.86)	0.59 (0.58–0.60)	0.60 (0.59–0.61)
Net Clinical and Mortality Outcome	49.0/61.5	44.35 (43.55–45.16)	0.59 (0.58–0.61)	0.61 (0.60–0.62)
**DOACs (** ***n*** ** = 456,792)**				
Primary Effectiveness Outcome	0.8/1.3	0.87 (0.72–1.06)	0.64 (0.53–0.77)	0.65 (0.53–0.79)
Primary Safety Outcome	1.7/1.3	1.92 (1.68–2.19)	1.39 (1.22–1.59)	1.36 (1.19–1.56)
Net Clinical Outcome	2.5/2.7	2.80 (2.51–3.12)	1.01 (0.91–1.13)	1.01 (0.90–1.13)
			**Cox proportional hazard model**	
			**Crude HR**	**Adjusted HR**
Death	37.0/61.1	41.68 (40.53–42.86)	0.54 (0.53–0.56)	0.59 (0.57–0.60)
Net Clinical and Mortality Outcome	37.3/61.5	42.44 (41.27–43.63)	0.54 (0.53–0.56)	0.59 (0.57–0.60)

*Abbreviations* Subdistribution hazard ratio (SHR); hazard ratio (HR); direct-acting oral anticoagulant (DOAC)

The results from the stratified analyses defined by the risk of stroke and bleeding and by antiplatelet use showed that although the point estimates for all outcomes were directionally consistent across the four subgroups, OAC use was associated with the lowest rate of the net clinical outcome in the strata containing residents with the highest bleeding and stroke risk (Supplemental Table 4.1–4.4). The associations of OAC use with all outcomes among antiplatelet users and non-users were generally consistent with the primary analyses (Supplemental Table 5.1–5.2).

## Discussion

Among US nursing home residents with atrial fibrillation, we found that OAC use was associated with a lower rate of developing either an ischemic stroke or a systemic embolism, a higher likelihood of developing an episode of intracranial or extracranial bleeding, and a lower net clinical and mortality risk compared with no use of OACs. Results of stratified analyses by OAC class among warfarin users and DOAC users in comparison with OAC non-users indicated that both anticoagulant classes were associated with a similar magnitude of benefit for ischemic stroke and systemic embolism prevention. Warfarin users, but not DOAC users, had a higher rate of the net clinical outcome versus OAC non-users.

### Comparison with previous clinical trials on effectiveness and safety of OAC use

To our knowledge, no clinical trials have directly compared outcomes of clinical relevance in OAC users versus non-users with atrial fibrillation in the nursing home setting. This is likely due in part to the advanced age and multimorbidity of the nursing home population, and the absence of a clear financial incentive or regulatory requirement to conduct trials of OACs in this population. Most recent trials have compared effectiveness and safety between warfarin and DOAC use in community-dwelling patients with atrial fibrillation, after earlier studies had clearly demonstrated the superiority of OAC use [11–15, 45].

The incidence of ischemic stroke or systemic embolism among older patients (mean age: 83 years) treated with OACs in our study (0.9% per-years) was comparable to the rate of ischemic stroke or systemic embolism observed in the warfarin arm of the Birmingham Atrial Fibrillation Treatment of the Aged Study (BAFTA) trial, which was conducted among adults aged ≥ 75 years (mean age: 82 years). Rates of hospitalization for bleeding events among OAC users in our study (2.3% per-year) were slightly higher than that observed among warfarin users in the BAFTA trial (1.9% per-year). This could be explained in part by differences in approaches to outcome ascertainment, since the BAFTA trial used brain imaging studies to define the presence of an intracranial hemorrhage, while our outcomes definitions were based on Medicare Part A claims.

In a meta-analysis of the warfarin arms of trials comparing warfarin against alternative thromboprophylaxis strategies, the rate of stroke or systemic embolism was higher (1.7% per-year), although this difference was diminished (1.2% per-year) when hemorrhagic stroke was removed from the outcome definition.^8^ The pooled rate of major bleeding was slightly higher (2.7% per-year) than the rate of bleeding hospitalizations in our study. One of the possible reasons for this difference is that trial enrollees were generally younger and healthier than the residents in our study, which may contribute to fewer fatal bleeding events that do not lead to hospitalization. Other than BAFTA, the other seven trials in the meta-analysis had patient populations with a lower mean age of 73 years or less, most were men, and patients in most of the trials (except in ROCKET-AF) had a lower prevalence of diabetes compared with our study population.[8,12–15].

### Comparison with previous comparative studies on effectiveness and safety of OAC use

In an observational study that used multiple data sources to compare outcomes between OAC users versus non-users among community-dwelling patients with atrial fibrillation, OAC use was associated with a lower risk of dying and stroke/systemic embolism but an increased risk of major bleeding [46]. We obtained directionally similar results in a nursing home population with higher magnitude associations between OAC use and mortality and bleeding, likely due to the older age and higher risk of developing clinical outcomes among nursing home than community dwelling residents [47]. Additionally, we observed an association of OAC use with lower incidence of mortality, compared with non-users, consistent with other observational studies that focused on nursing home populations with dementia [48, 49].

### Instrumental variable approach findings

In nursing home settings, the unexplained between-nursing home variation in medication prescribing practices is due to the multi-factorial effects of facility level characteristics and prescribing cultures [44, 50, 51]. However, no trials comparing OAC use versus non-use have been performed in nursing home settings and no prior observational study has compared the effectiveness and safety of OAC use versus non-use using an instrumental variable analysis. If assumptions are satisfied, this method theoretically adjusts for unobserved confounders to provide additional evidence regarding the robustness of the original study results. The results of our IV approach were directionally consistent with our primary findings, while differences in the magnitudes of association may suggest the potential existence of unobserved confounding, or heterogeneity of treatment effects for the subgroup of residents to whom the instrumental variable analyses apply versus the full population [52].

### Results between warfarin users and DOAC users

Prior head-to-head trials and observational studies have found a favorable net clinical outcome for DOACs versus warfarin [12–15, 53]. Our study indicated that both anticoagulant classes confer survival advantages and a lower risk of ischemic events compared with no treatment. Although both DOACs and warfarin users had higher rates of bleeding than non-users, consistent with previous observational studies and clinical trials, DOAC users appeared to have lower rates of bleeding than warfarin users and only warfarin had a negative net clinical outcome compared with no OAC use [10, 53–55].

### Who could benefit from OAC use?

An effective and acceptable OAC treatment plan requires a shared decision-making process that involves nursing home residents, care providers, and family members to balance tradeoffs in the use of OAC therapy to achieve goal-concordant care. To better resolve the dilemma of weighing the reduction of stroke risk against increased bleeding risk, we performed stratified analyses among nursing home residents who had varying levels of subsequent stroke and bleeding risk. OAC users who were not in the strata at highest risk of stroke (CHA_2_DS_2_-Vasc *≥* 7) had a negative net clinical outcome versus non-users, while those in the highest risk group had a comparable rate of net clinical outcomes. This finding suggests that OAC use would produce the largest net clinical outcome among older nursing home residents with atrial fibrillation who have the highest level of stroke risk. However, there was a sizable mortality advantage for OAC use across all subgroups defined by outcome risk and OAC type, which supports a potentially stronger case for OAC use among all nursing home residents with atrial fibrillation for whom extended survival is a goal of care. Beyond stroke and bleeding risk, decisions regarding OAC use and OAC selection should consider other factors such as monitoring requirements, anticoagulation reversal, and drug (and food) interactions. Although warfarin is easier to reverse, it has a greater potential for drug interactions. Extensive polypharmacy among nursing home residents and historical challenges maintaining warfarin within the therapeutic range have likely contributed to the shift from predominant warfarin to DOAC use in the NH setting [31, 56].

#### Study strengths and limitations

Our study provides national comparative effectiveness and safety data on OAC users versus non-users among U.S. nursing home residents with atrial fibrillation. We used the most recently available national dataset available to our research team which contained information from all Medicare/Medicaid certified nursing homes and comprehensive assessments for all nursing home residents. However, our dataset did not contain electronic health record nor laboratory, imaging, surgery, or autopsy data that most trials have used to define various clinical outcomes. Moreover, Part D claims do not indicate whether a drug was consumed, although adherence is less of a concern in nursing homes where medications are staff-administered. Our primary analyses were focused on OAC users vs. non-users. In stratified analyses, we compared DOAC initiators and warfarin initiators versus non-users separately, but we did not evaluate the effects of switching between different types of OACs during follow-up. We captured outcomes using Part A hospital claims and may have potentially underestimated the outcome rates for minor and fatal events that do not lead to hospitalization. Since the goals of care are likely different in older nursing home versus community-dwelling populations, our findings require cautious interpretation when used for informing decisions for populations residing outside of the nursing home setting.

## Conclusions

Among US nursing home residents aged ≥ 65 years with atrial fibrillation, OAC use was associated with a lower risk of ischemic stroke or systemic embolism and dying and a higher risk of intracranial or extracranial bleeding. Our results support the benefits of treatment with OACs to prevent ischemic strokes and increase longevity, while highlighting the need to weigh apparent benefits against elevated risk for bleeding with resident-centered shared decision-making.

### Electronic supplementary material

Below is the link to the electronic supplementary material.

Supplementary Material 1

## Data Availability

The datasets used and/or analyzed during the current study are available from the corresponding author on reasonable request.
