# Supplementary material for: To treat or not to treat: a comparative effectiveness analysis of oral anticoagulant outcomes among U.S. nursing home residents with atrial fibrillation
Source: BMC Geriatr. 2024 Jul 19;24:619. doi: 10.1186/s12877-024-05186-9 (PMC11264888; doi:10.1186/s12877-024-05186-9)

**Supplemental Materials Content List**

**Part 1 Technical Appendix**

**Part 2 Supplemental Tables**

Supplemental Table 1 Crude and adjusted subdistribution hazard ratios (SHR) for primary outcomes and hazard ratio (HRs) for secondary outcomes in Cox proportional hazard models comparing OAC users and non-users by using two-stage Instrumental variable (IV) model^*^

Supplemental Table 2 Strength of instrumental variables based on nursing home prescribing preference in OAC use

Supplemental Table 3.1 Estimated prevalence, crude and adjusted subdistribution hazard ratios (SHR) for primary outcomes and hazard ratios for secondary outcomes comparing warfarin users and OAC non-users among those whose index date were between 2011-2013 (N=225,063)

Supplemental Table 3.2 Estimated prevalence, crude and adjusted subdistribution hazard ratios (SHR) for primary outcomes and hazard ratios for secondary outcomes comparing DOAC users and OAC non-users among those whose index date were between 2011-2013 (N=214,274)

Supplemental Table 3.3 Estimated prevalence, crude and adjusted subdistribution hazard ratios (SHR) for primary outcomes and hazard ratios for secondary outcomes comparing warfarin users and OAC non-users among those whose index date were between 2014-2016 (N=242,220)

Supplemental Table 3.4 Estimated prevalence, crude and adjusted subdistribution hazard ratios (SHR) for primary outcomes and hazard ratios for secondary outcomes comparing DOAC users and OAC non-users among those whose index date were between 2014-2016 (N=242,518)

Supplemental Table 4.1 Estimated prevalence, crude and adjusted subdistribution hazard ratios (SHR) for primary outcomes and hazard ratios for secondary outcomes comparing OAC users and OAC non-users among those who had highest risk of stroke and highest risk of bleeding (N=51,304)

Supplemental Table 4.2 Estimated prevalence, crude and adjusted subdistribution hazard ratios (SHR) for primary outcomes and hazard ratios for secondary outcomes comparing OAC users and OAC non-users among those who had highest risk of stroke but not with highest risk of bleeding (N=30,101)

Supplemental Table 4.3 Estimated prevalence, crude and adjusted subdistribution hazard ratios (SHR) for primary outcomes and hazard ratios for secondary outcomes comparing OAC users and OAC non-users among those who had highest risk of bleeding but not with highest risk of stroke (N=183,676)

Supplemental Table 4.4 Estimated prevalence, crude and adjusted subdistribution hazard ratios (SHR) for primary outcomes and hazard ratios for secondary outcomes comparing OAC users and OAC non-users among those who had neither highest risk of stroke nor highest risk of bleeding (N=215,510)

Supplemental Table 5.1 Estimated prevalence, crude and adjusted subdistribution hazard ratios (SHR) for primary outcomes and hazard ratios for secondary outcomes comparing OAC users and OAC non-users among those who used antiplatelet at the same time (N=86,409)

Supplemental Table 5.2 Estimated prevalence, crude and adjusted subdistribution hazard ratios (SHR) for primary outcomes and hazard ratios for secondary outcomes comparing OAC users and OAC non-users among those who did not use antiplatelet at the same time (N=394,182)

Supplemental Table 6 Operational definitions of clinical conditions

**Part 3 Supplemental Figures**

Supplemental Figure 1: Study population selection for OAC users

Supplemental Figure 2: Study population selection for OAC non-users

Supplemental Figure 3: Distribution of instrumental variable

**Part 1 Technical Appendix**

**Instrumental Variable Rationale**

We conducted an instrumental variable analysis to investigate the robustness of the results of our main analysis to potential unmeasured confounding due to differences in characteristics of OAC users and non-users not captured by our data sources. An instrumental variable is sometimes referred to informally as a natural randomizer; it is a variable that affects which exposure group an individual is in but is not associated with the outcome directly or indirectly through any other causal path (the key assumptions of an instrumental variable analysis). By sorting individuals based on the instrument, if assumptions are satisfied, then both measured and unmeasured characteristics are balanced. In contrast, our primary analysis adjusts for a large set of observed potential confounders but does not address unmeasured confounding.

In our study, we used nursing home level OAC prescribing preference (i.e., historical propensity to use OACs for residents with atrial fibrillation) as an instrument. Instrumental variable analysis estimates a marginal effect, in contrast to the full population effect estimate from our primary analysis. This marginal effect can be interpreted as applying to the subgroup of individuals where there is likely to be the greatest equipoise about whether or not to treat, and these are the individuals for whom the instrument was most likely to determine their exposure status (i.e., residents who would have been treated in a high OAC prescribing nursing home but not in a low OAC prescribing nursing home). We restricted our instrumental variable analysis to residents in the highest and lowest quartiles of nursing home OAC prescribing preference, where the contrast in prescribing preference is sharpest. Therefore, differences in results observed between our main full population analysis and the instrumental variable analysis may be driven by a different association within the marginal subgroup of residents, or by adjustment for unmeasured confounding present in the main analysis.

**Instrumental Variable Analytic Approach**

**Step 1** (Estimating propensity scores): Instead of including many covariates in the model, we included a summary score (i.e., propensity score) to adjust for confounding effects.^1^ After performing the Cox proportional hazard regression models for the estimation of hazard ratios on the primary study outcomes, we identified several potential confounders that were not statistically significantly associated with both outcomes: rejected care, diagnosis of Alzheimer’s disease and related dementia, and polypharmacy and excluded them for further propensity score estimation. Then we used a multivariable logistic model to estimate a propensity score for each resident $i$ at each facility $j$:

$logit\left( P\left( T_{ij}=1 | \boldsymbol{Z}_{i} \right) \right)=\alpha_{0}+\sum_{j=1} \alpha_{j}Z_{ij}$,

where $T_{i}$ is the indicator for OAC use for patient $i$, and $\boldsymbol{Z}_{i}$ is a set of individual covariates including those statistically significantly associated with the primary study outcomes; age, sex, race/ethnicity, BMI, functional dependency, cognitive impairment, number of hospital admissions, history of falling, diagnosis of cancer, CHADs-VASc risk scores, ATRIA score, use of NSAIDs, use of statins, and use of antiplatelet therapy. Once the model was fitted, we estimated each propensity, $e_{ij}=logit^{-1}\left( \hat{\alpha}_{0}+\sum_{j=1} \hat{\alpha}_{j}Z_{ij} \right)$, where $logit^{-1}\left( \cdot\right)$ is the inverse logit function.

**Step 2** (Generating IV of nursing home prescribing preference): As similarly used in a previous study,^2^ we defined the nursing home level preference for prescribing OACs to residents as the instrumental variable (IV). We used a mixed effects logistic regression model to estimate the IV by using propensity score as the fixed effect to estimate the facility-level prescription preference as the facility random intercept in the model (difference between the expected and observed OAC prescribing probabilities for the nursing home).^3^ The fitted model is

$logit\left( T_{ij}=1 | e_{ij} \right)=\beta_{0}+b_{0j}+\beta_{1}e_{ij}$,

where $b_{0j}$ is a facility random intercept, $b_{0j} \sim N\left( 0, \tau^{2} \right)$ and $e_{ij}$ is the estimated propensity score by individual-level variables.

Once the facility random intercept was estimated, we assessed the distribution of the random facility intercept by histogram (Supplemental Figure 3). We then examined three cutoffs of the IV value: median, quartiles, and quintiles; we assigned each resident into each of categories according to their IV by creating three new variables.^2^ “IV-median” variable was a binary variable indicated the resident’s random intercept was above or below the median of all IVs. “IV-quartile” variable has four categories (0,1,2,3), indicated the resident’s random intercept sit in either below the lower quartile, between the lower quartile and median, higher than median and lower than the higher quartile or higher than the higher quartile. “IV-quintile” variable has five categories (0,1,2,3,4), indicated the resident’s random intercept position in the IV distribution classified by quintiles. Note that we limited the study population whose IV was in the lowest and highest categories of these three variables for subsequent analyses.

**Step 3** (Exploring plausibility of IV assumptions): We examined the assumptions of IV in all three cutoffs, IV-median, IV-quartile, and IV-quintile. To assess instrument strength, we estimated semi-partial R^2^ that represents the proportion of the variance of OAC use that could be explained by these IVs.^4^ We fitted the linear regression models with OAC use as the outcome and the predictors were IV-median, IV-quartile, IV-quintile respectively, with and without adjustment for the propensity score. We have run six models altogether. The estimate of the semi-partial R^2^ for each of the models for these three cutoffs (i.e., IV-median, IV-quartile, IV-quintile) were 2.2%, 4.6% and 5.2% accordingly. The semi-partial R^2^ between the models with and without propensity score showed similar results. As the semi-partial R^2^ could be interpreted the strength of the instrument that contributes to the prediction of the treatment. And these 2.2%-5.2% indicated relatively low or medium level of strength of instrumental variable.

In addition, we tested the independence assumption that the instrumental variable would be able to improve the balance between measured and unmeasured covariates. We fitted a multivariate linear regression model with OAC use as the outcome, with all covariates included in the propensity score included in the model as predictors. Then we pulled all of the diagnostic statistics to assess the influence of data points. Then, we used the following equation and the studentized residuals, number of study population to calculate the baseline Mahalanobis score. We used the mean value of Mahalanobis score in the study population as a reference balance score (2533.72). For each cutoff variable, we calculated their own mean Mahalanobis score. As an example of IV-median, we created two subsets of datasets as one included all residents whose IV was below the median, and another dataset included residents whose IV was above the median. We calculated the sum the total Mahalanobis score from the two subsets and divided over the number of total study population to obtain the mean Mahalanobis score. We compared the mean Mahalanobis score with IV-median cutoff with the reference balance score. We observed a decrease at -24.32%, indicating using IV-median has improved the balance between observed covariates in the study population, indirectly supported the potential improved balance of unobserved covariates between groups. For IV-quartile and IV-quintile cutoffs, comparing reductions of balance scores was done similarly as with IV-median: -49.26%, -53.14% respectively.

**Step 4** (2-stage IV models): We performed 2-stage least squares models in the survival context to estimate hazard ratios for primary and secondary outcomes comparing OAC use to non-use, with and without the propensity score, $e_{ij}$. At the 1-stage model, the logistic linear regression was fitted as follows; $logit\left( P\left( T_{ij}=1 | IV_{j}, e_{ij} \right) \right)=\gamma_{0}+\gamma_{1}IV_{j}+\gamma_{2}e_{ij}$. Then, we used the “predicted” value from the fitted model,  $\hat{T}_{ij}=logit^{-1}\left( \hat{\gamma}_{0}+\hat{\gamma}_{1}IV_{j}+\hat{\gamma}_{2}e_{ij} \right)$. At the 2-stage model, we fitted a Cox proportional hazards model with predicted OAC value, $\hat{T}_{ij}$, with and without adjusting for propensity score $e_{ij}$; $\lambda_{ij}\left( t \right)=\lambda_{0}\left( t \right)\exp\left( \beta_{1}\hat{T}_{ij}+\beta_{2}e_{ij} \right)$, where $\lambda_{0}\left( t \right)$ is the baseline hazard for non-user group and $\exp\left( \beta_{1} \right)$ is a hazard ratio of OAC user vs. non-user for each outcome.

To obtain valid standard error for the hazard ratio, a bootstrap method is applied in which we sampled subjects with replacements with the same sample size. With the sampled data, we fitted two-stage models and repeated 2,000 times.

**Interpretation of Instrumental Variable Analysis Results**

Point estimates for the instrumental variable analyses comparing OAC users and non-users were similar to the findings of our main analysis for the primary effectiveness outcome, primary safety outcome, and for the net clinical outcome. The protective association observed for OAC use with the mortality and net clinical and mortality outcome was stronger in the instrumental variable analysis than in the main analysis. This suggests that either 1) OAC users had a higher unmeasured risk of death in the main analysis and this was better adjusted for by the instrumental variable analysis, or 2) the mortality reduction benefit of OACs was greater within the subgroup of residents for whom there was the greatest uncertainty regarding whether to use OACs and for whom the nursing home’s prescribing preference was likely to have determined treatment status. We find some combination of these two explanations most plausible. Differences in observed characteristics depicted in Table 1 (e.g., hospital and SNF utilization at baseline) suggest potential greater mortality risk among new OAC user. Mortality benefits were largest in subgroup analyses among residents at the highest risk of stroke and bleeding (Supplemental Table 4.1), where clinical equipoise is likely to be greatest. In summary, we interpret the findings of our instrumental variable analysis as supportive of the robustness of the findings of our main analysis, and suggestive of a larger mortality benefit among the nursing home residents for whom there is the greatest uncertainty about whether or not to treat. Our results may reassure residents and their care teams who are opting for OAC use in such situations of clinical uncertainty, particularly when increased survival is a treatment goal.

References

1. Garrido MM, Kelley AS, Paris J, et al. Methods for Constructing and Assessing Propensity Scores. *Health Serv Res*. 2014;49(5):1701-1720. doi:10.1111/1475-6773.12182

2. Huybrechts KF, Gerhard T, Franklin JM, Levin R, Crystal S, Schneeweiss S. Instrumental variable applications using nursing home prescribing preferences in comparative effectiveness research. *Pharmacoepidemiol Drug Saf*. 2014;23(8):830-838. doi:10.1002/pds.3611

3. Wong GY, Mason WM. The Hierarchical Logistic Regression Model for Multilevel Analysis. *J Am Stat Assoc*. 1985;80(391):513-524. doi:10.2307/2288464

4. Brookhart MA, Rassen JA, Schneeweiss S. Instrumental variable methods in comparative safety and effectiveness research. *Pharmacoepidemiol Drug Saf*. 2010;19(6):537-554. doi:10.1002/pds.1908

**Supplemental Table 1 Crude and adjusted subdistribution hazard ratios (SHR) for primary outcomes and hazard ratio (HRs) for secondary outcomes in Cox proportional hazard models comparing OAC users and non-users by using two-stage Instrumental variable (IV) model^*^**

|  | **Two-stage IV Model^a^**  **(N=258,809)** | |
| --- | --- | --- |
|  | **Crude SHR^a^** | **Adjusted SHR** |
| **Primary Effectiveness Outcome** | 0.54 (0.38- 0.77) | 0.71 (0.47- 1.06) |
| **Primary Safety Outcome** | 1.87 (1.50- 2.33) | 1.86 (1.43- 2.43) |
| **Net Clinical Outcome** | 1.14 (0.94- 1.38) | 1.26 (1.00- 1.57) |
|  | **Two-stage IV Model^b^**  **(N=258,809)** | |
|  | **Crude HR** | **Adjusted HR** |
| **Death** | 0.29 (0.28- 0.31) | 0.41 (0.39- 0.43) |
| **Net Clinical and Mortality Outcome** | 0.30 (0.29- 0.32) | 0.42 (0.39- 0.44) |

^*^ The first stage is a logistic model where the outcome was observed OAC use, independent variables included the IV in quartiles and propensity score among residents who were classified in the highest and lowest IV quartiles. The second stage Fine and Gray models and Cox proportional hazards models included the predicted probabilities of OAC use estimated from the 1st-stage logistic model.

^b^ The adjusted model included the predicted probabilities of OAC use estimated from the 1^st^-stage logistic model and all covariates used for propensity score estimation.

^a^ These two-stage IV models were applied in Fine and Gray model context, by using death as the competing risk;

^b^ These two-stage IV models were applied in Cox proportional hazard model context

**Supplemental Table 2 Strength of instrumental variables based on nursing home prescribing preference in OAC use**

|  | **Unadjusted** | | | **Adjusted^*^** | | |
| --- | --- | --- | --- | --- | --- | --- |
|  | Risk Ratio | 95% CI | Semi-Partial R^2^ | Risk Ratio | 95% CI | Semi-Partial R^2^ |
| **IV-1** | 1.09 | 1.09- 1.09 | 2.03% | 1.09 | 1.09- 1.09 | 2.03% |
| **IV-2** | 1.14 | 1.13- 1.14 | 4.22% | 1.14 | 1.13- 1.14 | 4.22% |
| **IV-3** | 1.15 | 1.15- 1.15 | 4.75% | 1.15 | 1.14- 1.15 | 4.76% |

* Adjusted by propensity score

IV-1: by median; N=480,591

IV-2: by quartiles; only the highest and lowest quartiles: N=240,345

IV-3: by quintiles; only the highest and lowest quintiles: N=192,292

**Supplemental Table 3.1 Estimated prevalence, crude and adjusted subdistribution hazard ratios (SHR) for primary outcomes and hazard ratios for secondary outcomes comparing warfarin users and OAC non-users among those whose index dates were between 2011-2013 (N=225,063)**

|  | **% on warfarin users** | **Fine and Gray model** | |
| --- | --- | --- | --- |
|  |  | **Crude SHR** | **Adjusted SHR** |
| **Primary Effectiveness Outcome** | 1.2 | 0.67 (0.57- 0.78) | 0.68 (0.58- 0.79) |
| **Primary Safety Outcome** | 3.1 | 1.72 (1.55- 1.89) | 1.64 (1.47- 1.82) |
| **Net Clinical Outcome** | 4.3 | 1.20 (1.11- 1.31) | 1.18 (1.08- 1.28) |
|  |  | **Cox proportional hazard model** | |
|  |  | **Crude HR** | **Adjusted HR** |
| **Death** | 52.2 | 0.54 (0.53- 0.55) | 0.56 (0.54- 0.57) |
| **Net Clinical and Mortality Outcome** | 53.3 | 0.55 (0.54- 0.56) | 0.56 (0.55- 0.58) |

**Supplemental Table 3.2 Estimated prevalence, crude and adjusted subdistribution hazard ratios (SHR) for primary outcomes and hazard ratios for secondary outcomes comparing warfarin users and OAC non-users among those whose index dates were between 2011-2013 (N=214,274)**

|  | **% on DOAC users** | **Fine and Gray model** | |
| --- | --- | --- | --- |
|  |  | **Crude SHR** | **Adjusted SHR** |
| **Primary Effectiveness Outcome** | 1.3 | 0.80 (0.59- 1.09) | 0.77 (0.56- 1.04) |
| **Primary Safety Outcome** | 3.0 | 1.65 (1.34- 2.03) | 1.57 (1.27- 1.94) |
| **Net Clinical Outcome** | 4.2 | 1.23 (1.04- 1.47) | 1.17 (0.98- 1.40) |
|  |  | **Cox proportional hazard model** | |
|  |  | **Crude HR** | **Adjusted HR** |
| **Death** | 45.9 | 0.49 (0.47- 0.52) | 0.54 (0.51- 0.56) |
| **Net Clinical and Mortality Outcome** | 46.9 | 0.50 (0.48- 0.53) | 0.54 (0.51- 0.57) |

**Supplemental Table 3.3 Estimated prevalence, crude and adjusted subdistribution hazard ratios (SHR) for primary outcomes and hazard ratios for secondary outcomes comparing warfarin users and OAC non-users among those whose index dates were between 2014-2016 (N=242,220)**

|  | **% on warfarin users** | **Fine and Gray model** | |
| --- | --- | --- | --- |
|  |  | **Crude SHR** | **Adjusted SHR** |
| **Primary Effectiveness Outcome** | 0.6 | 0.63 (0.49- 0.80) | 0.68 (0.53- 0.88) |
| **Primary Safety Outcome** | 2.0 | 2.13 (1.84- 2.46) | 2.09 (1.80- 2.44) |
| **Net Clinical Outcome** | 2.7 | 1.36 (1.20- 1.54) | 1.40 (1.23- 1.59) |
|  |  | **Cox proportional hazard model** | |
|  |  | **Crude HR** | **Adjusted HR** |
| **Death** | 42.7 | 0.65 (0.63- 0.67) | 0.67 (0.64- 0.69) |
| **Net Clinical and Mortality Outcome** | 42.9 | 0.66 (0.64- 0.68) | 0.67 (0.65- 0.69) |

**Supplemental Table 3.4 Estimated prevalence, crude and adjusted subdistribution hazard ratios (SHR) for primary outcomes and hazard ratios for secondary outcomes comparing DOAC users and OAC non-users among those whose index dates were between 2014-2016 (N=242,518)**

|  | **% on DOAC users** | **Fine and Gray model** | |
| --- | --- | --- | --- |
|  |  | **Crude SHR** | **Adjusted SHR** |
| **Primary Effectiveness Outcome** | 0.6 | 0.67 (0.52- 0.86) | 0.71 (0.55- 0.92) |
| **Primary Safety Outcome** | 1.3 | 1.58 (1.33- 1.88) | 1.58 (1.32- 1.89) |
| **Net Clinical Outcome** | 1.9 | 1.11 (0.96- 1.28) | 1.14 (0.99- 1.32) |
|  |  | **Cox proportional hazard model** | |
|  |  | **Crude HR** | **Adjusted HR** |
| **Death** | 34.3 | 0.60 (0.58- 0.63) | 0.64 (0.62- 0.67) |
| **Net Clinical and Mortality Outcome** | 34.4 | 0.61 (0.59- 0.63) | 0.64 (0.62- 0.67) |

**Supplemental Table 4.1 Estimated prevalence, crude and adjusted subdistribution hazard ratios (SHR) for primary outcomes and hazard ratios for secondary outcomes comparing OAC users and OAC non-users among those who had highest risk of stroke and highest risk of bleeding (N=51,304)**

|  | **% on OAC users** | **Fine and Gray model** | |
| --- | --- | --- | --- |
|  |  | **Crude SHR** | **Adjusted SHR** |
| **Primary Effectiveness Outcome** | 1.0 | 0.71 (0.50- 1.01) | 0.67 (0.47- 0.95) |
| **Primary Safety Outcome** | 2.2 | 1.31 (1.03- 1.68) | 1.27 (0.98- 1.65) |
| **Net Clinical Outcome** | 3.2 | 1.03 (0.84- 1.26) | 0.98 (0.80- 1.21) |
|  |  | **Cox proportional hazard model** | |
|  |  | **Crude HR** | **Adjusted HR** |
| **Death** | 50.3 | 0.56 (0.54- 0.59) | 0.57 (0.54- 0.60) |
| **Net Clinical and Mortality Outcome** | 50.8 | 0.57 (0.54- 0.60) | 0.58 (0.55- 0.61) |

**Supplemental Table 4.2 Estimated prevalence, crude and adjusted subdistribution hazard ratios (SHR) for primary outcomes and hazard ratios for secondary outcomes comparing OAC users and OAC non-users among those who had highest risk of stroke but not with highest risk of bleeding (N=30,101)**

|  | **% on OAC users** | **Fine and Gray model** | |
| --- | --- | --- | --- |
|  |  | **Crude SHR** | **Adjusted SHR** |
| **Primary Effectiveness Outcome** | 1.7 | 0.79 (0.58- 1.08) | 0.73 (0.53- 1.01) |
| **Primary Safety Outcome** | 2.3 | 1.68 (1.27- 2.23) | 1.75 (1.29- 2.38) |
| **Net Clinical Outcome** | 3.9 | 1.14 (0.93- 1.41) | 1.11 (0.89- 1.38) |
|  |  | **Cox proportional hazard model** | |
|  |  | **Crude HR** | **Adjusted HR** |
| **Death** | 44.2 | 0.56 (0.53- 0.60) | 0.58 (0.54- 0.61) |
| **Net Clinical and Mortality Outcome** | 44.8 | 0.57 (0.53- 0.60) | 0.58 (0.55- 0.62) |

**Supplemental Table 4.3 Estimated prevalence, crude and adjusted subdistribution hazard ratios (SHR) for primary outcomes and hazard ratios for secondary outcomes comparing OAC users and OAC non-users among those who had highest risk of bleeding but not with highest risk of stroke (N=183,676)**

|  | **% on OAC users** | **Fine and Gray model** | |
| --- | --- | --- | --- |
|  |  | **Crude SHR** | **Adjusted SHR** |
| **Primary Effectiveness Outcome** | 0.8 | 0.70 (0.57- 0.85) | 0.71 (0.58- 0.86) |
| **Primary Safety Outcome** | 2.5 | 1.70 (1.51- 1.91) | 1.59 (1.41- 1.80) |
| **Net Clinical Outcome** | 3.2 | 1.27 (1.15- 1.40) | 1.22 (1.10- 1.36) |
|  |  | **Cox proportional hazard model** | |
|  |  | **Crude HR** | **Adjusted HR** |
| **Death** | 48.9 | 0.59 (0.58- 0.61) | 0.59 (0.58- 0.61) |
| **Net Clinical and Mortality Outcome** | 49.5 | 0.60 (0.58- 0.61) | 0.60 (0.58- 0.61) |

**Supplemental Table 4.4 Estimated prevalence, crude and adjusted subdistribution hazard ratios (SHR) for primary outcomes and hazard ratios for secondary outcomes comparing OAC users and OAC non-users among those who had neither highest risk of stroke nor highest risk of bleeding (N=215,510)**

|  | **% on OAC users** | **Fine and Gray model** | |
| --- | --- | --- | --- |
|  |  | **Crude SHR** | **Adjusted SHR** |
| **Primary Effectiveness Outcome** | 0.9 | 0.60 (0.51- 0.71) | 0.66 (0.56- 0.77) |
| **Primary Safety Outcome** | 2.3 | 1.92 (1.73- 2.13) | 1.91 (1.71- 2.15) |
| **Net Clinical Outcome** | 3.1 | 1.20 (1.10- 1.31) | 1.25 (1.14- 1.37) |
|  |  | **Cox proportional hazard model** | |
|  |  | **Crude HR** | **Adjusted HR** |
| **Death** | 39.8 | 0.57 (0.56- 0.59) | 0.61 (0.59- 0.62) |
| **Net Clinical and Mortality Outcome** | 40.4 | 0.58 (0.56- 0.59) | 0.61 (0.60- 0.63) |

**Supplemental Table 5.1 Estimated prevalence, crude and adjusted subdistribution hazard ratios (SHR) for primary outcomes and hazard ratios for secondary outcomes comparing OAC users and OAC non-users among those who used antiplatelet at the same time (N=86,409)**

|  | **% on OAC users** | **Fine and Gray model** | |
| --- | --- | --- | --- |
|  |  | **Crude SHR** | **Adjusted SHR** |
| **Primary Effectiveness Outcome** | 1.3 | 0.81 (0.65- 1.01) | 0.89 (0.72- 1.11) |
| **Primary Safety Outcome** | 2.5 | 1.56 (1.33- 1.83) | 1.57 (1.33- 1.86) |
| **Net Clinical Outcome** | 3.8 | 1.19 (1.06- 1.32) | 1.25 (1.09- 1.42) |
|  |  | **Cox proportional hazard model** | |
|  |  | **Crude HR** | **Adjusted HR** |
| **Death** | 45.8 | 0.60 (0.58- 0.62) | 0.62 (0.60- 0.64) |
| **Net Clinical and Mortality Outcome** | 46.5 | 0.60 (0.58- 0.63) | 0.63 (0.60- 0.65) |

**Supplemental Table 5.2 Estimated prevalence, crude and adjusted subdistribution hazard ratios (SHR) for primary outcomes and hazard ratios for secondary outcomes comparing OAC users and OAC non-users among those who did not use antiplatelet at the same time (N=394,182)**

|  | **% on OAC users** | **Fine and Gray model** | |
| --- | --- | --- | --- |
|  |  | **Crude SHR** | **Adjusted SHR** |
| **Primary Effectiveness Outcome** | 0.8 | 0.62 (0.55- 0.71) | 0.63 (0.55- 0.72) |
| **Primary Safety Outcome** | 2.3 | 1.78 (1.64- 1.92) | 1.74 (1.59- 1.89) |
| **Net Clinical Outcome** | 3.1 | 1.20 (1.12- 1.28) | 1.19 (1.11- 1.28) |
|  |  | **Cox proportional hazard model** | |
|  |  | **Crude HR** | **Adjusted HR** |
| **Death** | 43.9 | 0.57 (0.56- 0.58) | 0.59 (0.58- 0.60) |
| **Net Clinical and Mortality Outcome** | 44.5 | 0.57 (0.56- 0.58) | 0.60 (0.59- 0.61) |

**Supplemental table 6 Operational definitions of clinical conditions**

| **Clinical Condition** | **ICD-9 CM Codes** | **ICD-10 CM Codes^*^** |
| --- | --- | --- |
| Atrial fibrillation/flutter^1,^ | 42731, 42732 | I480, I481, I482, I483, I484, I4891, I4892 |
| Valvular disease^2^ | 33400, 99602, 99661, V433, V422, 3979, 3971, 3970, 3969, 3968, 3963, 3962, 3961, 3960, 3959, 3952, 3951, 3950, 3949, 3942, 3941, 3940, 7467, 7466, 7465, 7464, 7463, 7462, 7461, 74609, 74602, 74601, 74600, 4243, 4242, 4241, 4240 | I050, I051, I052, I058, I059, I069, I068, I062, I061, I060, I080, I088, I089, I0989, I091, I083, I082, I081, I079, I078, I072, I071, I070, A1884, I340. I341, I342, I348, I349, I350, I351, I352, I358, I359, I360, I361, I362, I368, I370, I371, I372, I378, I379, I38, I39, M3211, Q209, Q220 Q221, Q222, Q223, Q224, Q225, Q226, Q228, Q229, Q230, Q231, Q232, Q234, Z953, Z952, Z954, T8201XA, T8201XD, T8201XS, T8202XA, T8202XD, T8202XS, T8203XA, T8203XD, T8203XS, T8209XA, T8209XD, T8209XS, T826XXA, T826XXD, T826XXS, |
| Ischemic stroke^3^ | 43301, 43311, 43321, 43331, 43381, 43391, 43401, 43411, 43491, 436 | I6302, I6312, I6322, I63239, I63232, I63231, I63139, I63132, I63131, I63039, I63032, I63031, I63011, I63012, I63019, I63111, I63112, I63119, I63211, I63212, I63219, I6359, I6319, I6309, I6329, I6320, I6310, I6300, I6330, I63311, I63312, I63319, I63321, I63322, I63329, I63331, I63332, I63339, I63341, I63342, I63349, I6339, I636, I6349, I63449, I63442, I63441, I63439, I69432, I69431, I63429, I63422, I63421, I63419, I63412, I63411, I63430, I6350, I63511, I63512, I63519, I63521, I63529, I63531, I63532, I63539, I63541, I63542, I63549, I6359, I638, I639, I6789 |
| Intracranial hemorrhage^4^ | 430, 431, 4320, 4321, 4329 | I609, I608, I607, I606, I6052, I6051, I6050, I604, I6032, I6031, I6030, I6022, I6021, I6020, I6012, I6011, I6010, I6002, I6001, I6000, I610, I611, I612, I613, I614, I615, I616, I618, I619, I621, I6200, I6201, I6202, I6203, I629 |
| Extracranial bleeding^4^ | In primary position alone:  5310, 5312, 5314, 5316, 5320, 5322, 5324, 5326, 5330, 5332, 5334, 5336, 5340, 5342, 5344, 5346, 53501, 53511, 53521, 53531, 53541, 53551, 53561, 53783, 4560, 45620, 5307, 53082, 5780, 4552, 4555, 4558, 56202, 56203, 56212, 56213, 56881, 5693, 56985, 5781, 5789, 59381, 5997, 6238, 6262, 6266, 4230, 4590, 56881, 7191, 7847, 7848, 7863  In primary position, with above code in secondary position:  5311, 5313, 5315, 5317, 5319, 5321, 5323, 5325, 5327, 5329, 5331, 5333, 5335, 5337, 5339, 5341, 5343, 5345, 5347, 5349, 53500, 53510, 53520, 53530, 53540, 53550, 53560, 455, 56200, 56201, 56210, 56211, 5301, 2800, 2851, 2859, 79092 | In primary position alone:  K252, K254, K256, K260, K262, K264, K266, K270, K272, K274, K276, K280, K282, K284, K286, K2901, K2931, K2941, K2951, K2961, K2921, K2971, K2981, K2991, K31811, I8501, I8511, K226, K228, K920, K648, K643, K642, K641 K640, K5711, K5751, K5753, K5741, K5713, K5701, K5791, K5731, K5793, K5781, K5733, K5721, K661, K625, K5521, K921, K922, N280, R310, R311, R312, R319, N898, N920, N921, I312, R58, M2500, M25011, M25012, M25019, M25021, M25022, M25029, M25031, M25032, M25039, M25041, M25042, M25049, M25051, M25052, M25059, M25061, M25062, M25069, M25071, M25072, M25073, M25074, M25075, M25076, M2508, R040, R041, R042, R0481, R0489, R049  In primary position, with above code in secondary position:  K251, K253, K255, K257, K259, K261, K263, K265, K267, K269, K271, K273, K275, K277, K279, K281, K283, K285, K287, K289, K2900, K2930, K2960, K2920, K2930, K2970, K2980, K640, K641, K642, K643, K644, K645, K648, K649, K5750, K5710, K5752, K5740, K5712, K5700, K5730, K5790, K5792, K5780, K5732, K5720, K210, K209, K208, K200, D800, D62, D649, R791 |
| Chronic renal insufficiency^5^ | 582, 583, 585, 586, 587 | M3218, M3214, M3504, N050, N051, N052, N053, N054, N055, N056, N057, N058, N059, N060, N061, N062, N063, N064, N065, N066, N067, N068, N069, N070, N071, N072, N073, N074, N075, N076, N077, N078, N079, N08, N140, N142, N144, N150, N158, N159, N171, N16, N170, N172, N178, N179, N181, N182, N183, N184, N185, N186, N189, N19, N261, N269 |
| Acute myocardial infarction^6^ | 41001, 41011, 41021, 41031, 41041, 41051, 41061, 41071, 41081, 41091 | I2109, I220, I2102, I2101, I2119, I221, I2111, I228, I2129, I214, I2121, I222, I229, I213 |
| Venous thromboembolism^7^ | 41511, 41519, 45111, 45119, 4512, 4519, 4531, 4532, 4534, 45341, 45342, 4538, 4539 | I803, I809, I821, I82220, I82401, I82402, I82403, I82409, I84211, I82412, I82413, I82419, I82421, I82422, I82423, I82429, I82431, I82432, I82433, I82439, I82441, I82442, I82443, I82449, I82491, I82492, I82493, I82499, I824Y1, I824Y2, I824Y3, I824Y9, I824Z1, I824Z2, I824Z3, I824Z9, I82210, I82290, I82601, I82602, I82603, I82609, I82611, I82612, I82613, I82619, I82621, I82622, I82629, I82890, I82891, I8290, I82A11, I82A12, I82A13, I82A19, I82A21, I82A22, I82A23, I82A29, I82B11, I82B12, I82B13I82B13, I82B19, I82B21, I82B22, I82B23, I82B29, I82C11, I82C12, I82C13, I82C19 |
| Systemic embolism^2^ | 444, 445 | I74 |
| Pneumonia^8^ | 480, 481, 482, 485, 486, 4870 | J120, J121, J122, J123, J1281, J1289, J129, J181, J13, J14, J150, J151, J152, J15211, J15212, J1529, J153, J154, J155, J156, J158, J159, J180, J188, J189, J129, J1108, J1100, J1008, J1001, J1000 |
| Chronic obstructive pulmonary disease^9^ | In primary position alone:  49121, 49122, 4918, 4919 4928, 49320, 49321, 49322, 496  In primary position, with above code in secondary position:  51881, 51882, 51884, 7991 | In primary position alone:  J441J J440, J418, J42, J439, J438, J432, J431, J430, J449  In primary position, with above code in secondary position:  J9600, J9601, J9602, J9690, J9691, J9692, J80, J9620, 9621, J9622, R092 |
| *ICD-9 CM code based algorithms were converted to ICD-10 CM codes using the 2016 General Equivalence Mappings available from the Centers for Medicare and Medicaid Services at <https://www.cms.gov/Medicare/Coding/ICD10/2016-ICD-10-CM-and-GEMs.html> | | |

**Part 3: Supplemental Figure 1: Study population selection for OAC users**


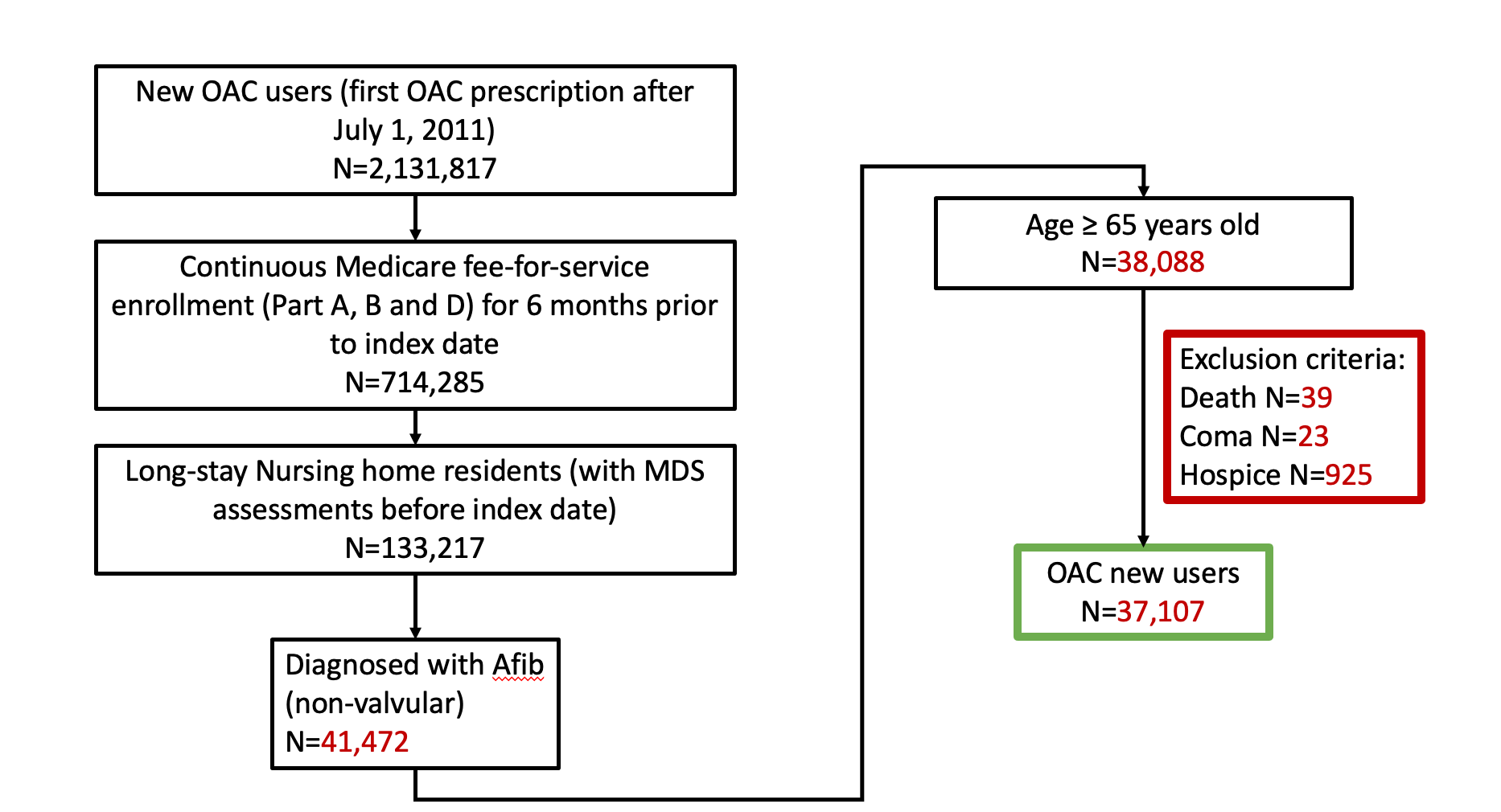


**Part 3: Supplemental Figure 2: Study population selection for OAC non-users**


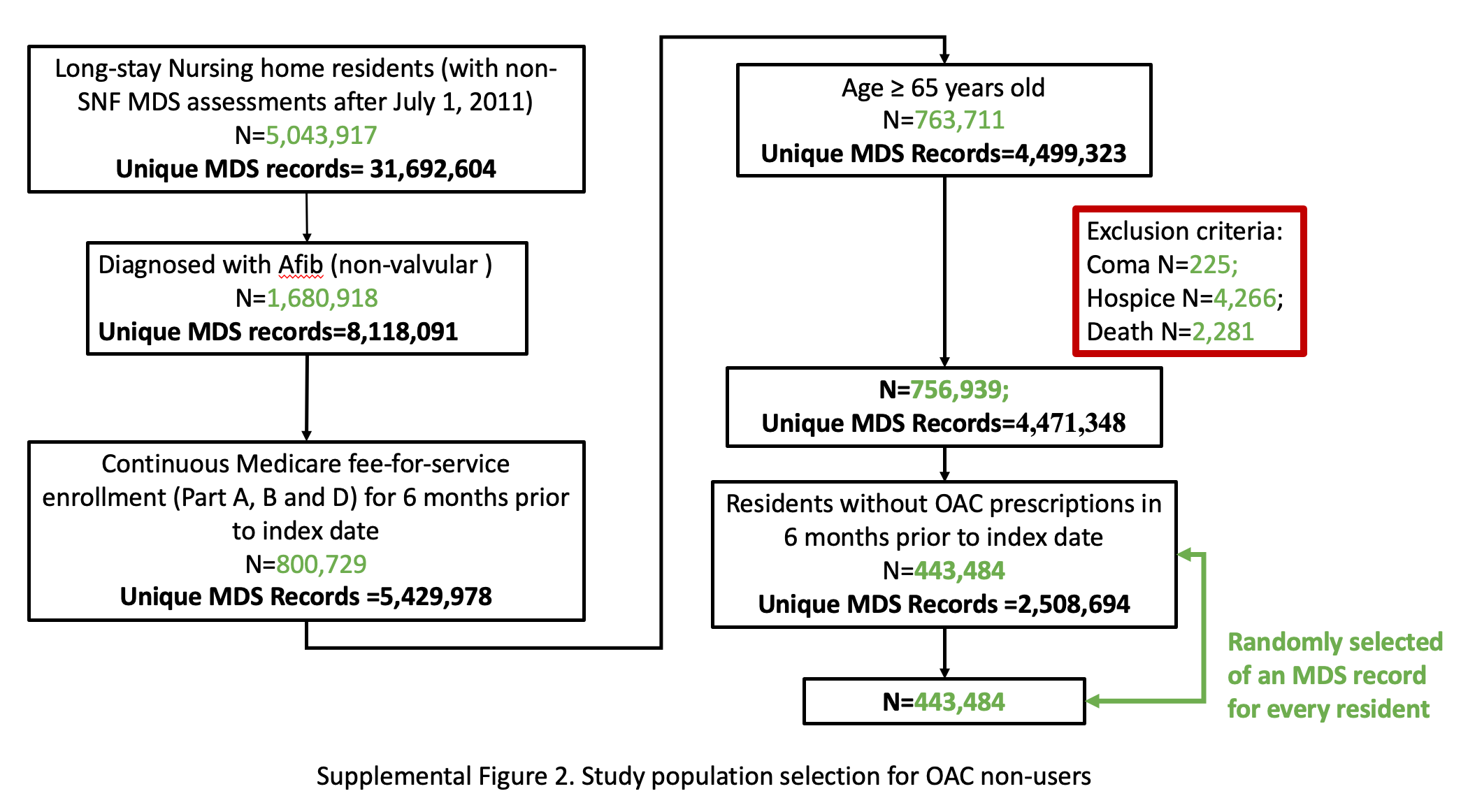


**Part 3: Supplement figure 3: Distribution of instrumental variable**


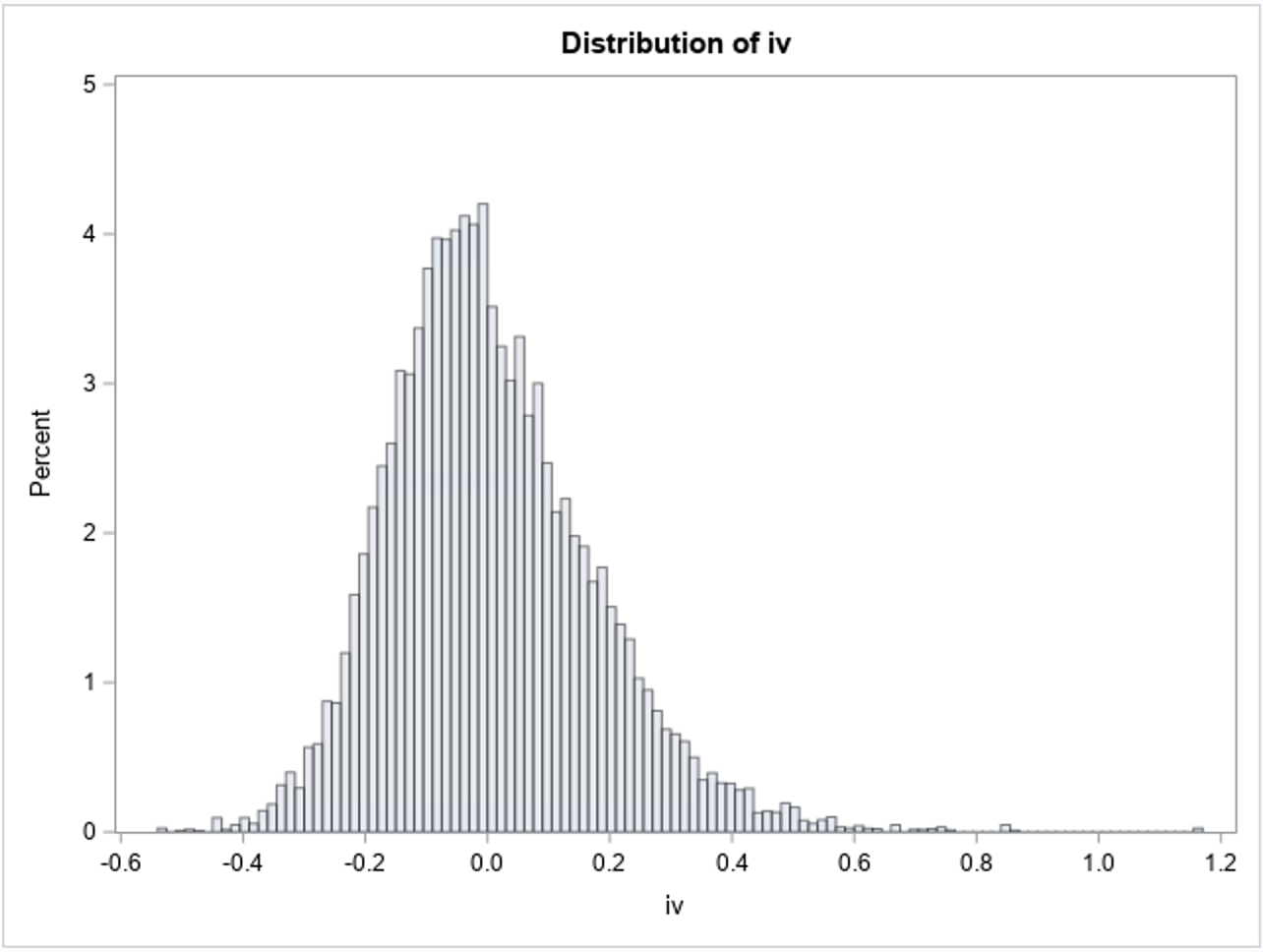

Supplement: Supplementary file 1 — Supplementary Material 1 [file 12877_2024_5186_MOESM1_ESM.docx]
